# Recycling waste via insect agriculture: Frass impacts on soil and plant health

**DOI:** 10.1002/jeq2.70089

**Published:** 2025-09-25

**Authors:** Helen C. S. Amorim, Amanda J. Ashworth, Thomas F. Ducey, Valerie B. Brewer‐Gunsaulis, Gerson L. Drescher, Phillip R. Owens, Alana H. Patterson, Giovanna De Blasis, Iris van Straaten

**Affiliations:** ^1^ Department of Crop, Soil, and Environmental Sciences University of Arkansas Fayetteville Arkansas USA; ^2^ USDA‐ARS Poultry Production and Product Safety Research Unit Fayetteville Arkansas USA; ^3^ USDA‐ARS Coastal Plain Soil, Water and Plant Conservation Research Florence South Carolina USA; ^4^ Tyson Foods Inc. Springdale Arkansas USA; ^5^ USDA‐ARS Dale Bumpers Small Farms Research Center Booneville Arkansas USA; ^6^ Protix B.V. Hertogenbosch The Netherlands

## Abstract

Frass—the main by‐product of insect rearing for animal feed—is emerging as a promising soil amendment and plant growth promoter. However, basic agronomic information is lacking and prevents frass’ widespread use as a biofertilizer. This study assessed impacts of black soldier fly (*Hermetia illucens* L. [Diptera: Stratiomyidae]) frass on soil fertility, crop growth, and quality compared to poultry litter (PL). Irrigated and non‐irrigated soybean (*Glycine max* L. Merr.) and non‐irrigated switchgrass (*Panicum virgatum* L.) plots received either PL (5.6 Mg ha^−1^), low frass rate (LF; 5.6 Mg ha^−1^), high frass rate (HF; 11.2 Mg ha^−1^), besides the unamended control (CT). In general, soil nutrients and enzymes at the soil surface (0–15 cm) were unaffected by soil amendments. Irrigated‐HF soybean had 7% higher grain P concentration than non‐irrigated‐HF, and 13% greater P concentration than the non‐irrigated CT. Additionally, HF increased K concentration in switchgrass by 25% relative to the CT. HF reduced soybean leaf damage by 35% and 48% relative to the non‐irrigated CT and PL‐irrigated plots, illustrating for the first time frass’ potential to enhance plant resistance to herbivory, likely owing to the presence of chitin. LF had 2–4 times greater nutrient use efficiency than HF and PL in organic soybean and switchgrass systems, reflective of similar yields despite lower nutrient inputs. These findings provide foundational knowledge for frass utilization as an organic fertilizer and biostimulant, closing nutrient loops through waste recovery during insect rearing, and supporting the development of an emerging sustainable industry.

## Core Ideas

- Insect manure “frass” is a novel organic fertilizer that can close nutrient loops through waste nutrient recovery.
- Poultry litter had up to 12 times higher heavy metals and potentially toxic elements than frass.
- High frass (11.2 Mg ha^−1^) increased P in soybean grains and K in switchgrass biomass compared to the control.
- High frass reduced soybean leaf damage by 38%–45% relative to the control and poultry litter.
- Two to four times higher nutrient use efficiency and enhanced plant defense show frass as a multiuse organic amendment.

## Plain Language Summary

As insect farming expands worldwide, insect manure—frass—production increases, and proper waste management is needed to support this growing industry and the upcycling of this enriched material. Scientists and industry stakeholders partnered in this research to test the frass of black soldier fly larvae, and understand how frass improves soils, plant growth, and grains and forage production compared to poultry litter, aiming to support frass utilization as an organic fertilizer. Frass not only increased essential nutrients in plants but also helped protect soybean leaves from pest insects, acting like a natural pesticide. Moreover, a lower frass application rate resulted in similar crop yields with much less nutrient input, showcasing frass efficiency as a nutrient source. These findings support frass use as a sustainable, eco‐friendly solution for farmers looking to improve their soils, crop production, and reduce reliance on traditional fertilizers.

**Abbreviations**
AcP: acid phosphatase
ADF: acid‐digestible fiber
BG: β‐glucosidase
BSF: black soldier fly
CT: control
EC: electrical conductivity
FDA: fluorescein diacetate
HF: high frass rate
LF: low frass rate
NAG: *N*‐acetyl‐β‐d‐glucosaminidase
NDF: neutral‐digestible fiber
NUE: nutrient use efficiency
PFP: partial factor productivity
PL: poultry litter
SOC: soil organic C
TC: total C

## 1 INTRODUCTION

Modern agriculture is tasked with increasing systems’ productivity to feed 9.8 billion people by 2050 (Food and Agriculture Organization of the United Nations [FAO], 2018) while minimizing social and environmental impacts. Insect farming or “ministock” is emerging as a more sustainable protein industry (Morales‐Ramos et al., 2024) as insects require 2–14 times less land and five times less water on an edible protein basis, with greater feed conversion efficiency (Cadinu et al., 2020; van Huis & Oonincx, 2017) and lower environmental footprint than conventional livestock (Oonincx et al., 2010). Additionally, commercially important insect species, such as black soldier fly [BSF; *Hermetia illucens* L. (Diptera: Stratiomyidae)], can be fed food and agricultural waste, yielding protein for animal feed while reducing landfill waste accumulation (Henault‐Ethier et al., 2024; Tabass et al., 2016).

As insect agriculture expands, projections show that 5 million tonnes of insect manure, or frass, will be produced by 2033 only in North America (Henault‐Ethier et al., 2024). Frass is the mixture of excreta, feed, and molted skins resulting from insect rearing, enriched in macronutrients (e.g., N, P, K, Ca, and Mg) and therefore a promising soil amendment and plant growth promoter (Amorim et al., 2024; Ashworth et al., 2025; K. Robinson et al., 2024). Benefits from insect frass include increased soil fertility and nutrient uptake by crops, enhanced soil microbial activity, and greater seed germination and plant biomass (Awad et al., 2024; Beesigamukama et al., 2022; Karkanis et al., 2024; Menino et al., 2021; Poveda, 2021) compared to commercial and other organic fertilizer sources (Beesigamukama et al., 2020; Carroll et al., 2023; Houben et al., 2020). Additionally, frass contains chitin and volatile organic compounds, which can enhance plant resistance to herbivory and trigger insect repellency (Barragán‐Fonseca et al., 2022), potentially reducing pest damage. Yet, these properties remain largely untested in the field, and frass from prominent insect protein industries is currently underutilized or wasted due to the lack of basic agronomic information to support frass use by farmers (K. Robinson et al., 2024).

Pioneering fieldwork from Ashworth et al. (2025) showed that soil fertility metrics improved substantially after 2 years of frass applications in forage systems. However, no differences in overall soil health, CO_2_ efflux, or forage yields and quality were observed among low or high frass rates, ammonium nitrate, or poultry litter (PL). The lack of response on biomass production contrasts with the general trends in literature, although these positive impacts of frass on plant growth were demonstrated in controlled environment conditions, soils with optimum nutrient availability, and/or applying higher frass rates (Houben et al., 2020; Menino et al., 2021; Tanga et al., 2022). Nevertheless, Ashworth et al. (2025) showed that frass impacts forage production and environmental quality similarly to other fertilizer sources, and pointed out research gaps to be addressed to optimize frass use. Specifically, frass land application rates need adjustments according to the soil‐cropping system and may not follow traditional recommendation for manures, owing to the different concentrations of total, organic, and plant‐available nutrients (Amorim et al., 2024; Ashworth, Chastain, et al., 2020).

Finding cost‐effective and environmentally friendly nutrient sources is key for sustainable intensification in agriculture. Frass is a multi‐nutrient source resulting from the upcycling of agrifood waste, which can help advance low‐input and organic agriculture and enhance the system's One Health (i.e., healthy soils‐plants‐animals‐humans). Therefore, this study aims to identify the impacts of BSF larvae frass on soil health, plant growth, crop yields and nutritional value, and foliar damage (as a measure of enhanced plant defense) compared to PL, to further allow frass valorization as an organic soil amendment. We hypothesized that (i) row crops and pasture receiving BSF larvae frass will have greater soil fertility, grain/biomass yields and quality, and resistance to biotic (pests) stress than PL, and (ii) these improvements will be proportional to land application rates (low frass rate [LF] and high frass rate [HF], 5.6 and 11.2 Mg ha^−1^, respectively) and will vary according to cropping system (soybean [*Glycine max* L. Merr.] and switchgrass [*Panicum virgatum* L.]).

## 2 MATERIALS AND METHODS

### 2.1 Site description and experimental setup

This study was conducted on organic certified land at the USDA‐ARS Dale Bumpers Small Farms Research Center in Booneville, AR. The study site is situated in the Arkansas Valley and Ridges province, with soil classified as Leadvale silt loam (fine‐silty, siliceous, semiactive, and thermic Typic Fragiudults) with a fragipan at a depth of 14–97 cm (Soil Survey Staff, 2022). Total annual precipitation in the region is 1220 mm, and average annual air temperature ranges between 15.8°C and 17.4°C (2014–2023; NOAA, 2024).

Two field trials were established in a completely randomized design: one in soybean (*Glycine max* L. Merr.) and the other in an established switchgrass (*Panicum virgatum* L.) system. Experimental plots of 3 m × 6.1 m were established on April 23, 2024, on a uniform 2% slope. For soybean, fixed effects were irrigation (irrigated and non‐irrigated) in the whole plot, and soil amendments (PL; 5.6 Mg ha^−1^], LF and HF [5.6 and 11.2 Mg ha^−1^, respectively], and an unamended control [CT]) as the split‐plot treatments (two main effects × four treatments × three repetitions = 24 plots). The PL application rate was defined to provide 168 kg N ha^−1^ (Ashworth et al., 2022), and frass rates mirrored those of PL on a mass basis. Soil amendments were surface‐applied in both systems. As for switchgrass, whole plots were soil amendments, totaling 12 plots of 3 m × 3 m in a 1% slope (four treatments × three repetitions). Soil amendments were applied on April 29, 2024, in both systems (soybean and switchgrass).

**Core Ideas**

- Insect manure “frass” is a novel organic fertilizer that can close nutrient loops through waste nutrient recovery.
- Poultry litter had up to 12 times higher heavy metals and potentially toxic elements than frass.
- High frass (11.2 Mg ha^−1^) increased P in soybean grains and K in switchgrass biomass compared to the control.
- High frass reduced soybean leaf damage by 38%–45% relative to the control and poultry litter.
- Two to four times higher nutrient use efficiency and enhanced plant defense show frass as a multiuse organic amendment.

### 2.2 Frass and poultry litter production

BSF eggs were obtained from the Protix B.V. (Bergen op Zoom, The Netherlands) production colony and shipped to the TyPro research lab (Springdale, AR). Ovisites containing BSF eggs were placed into plastic containers (11 cm × 32 cm × 21 cm) covered with mesh and sealed with an aerated lid. Containers were placed into incubating chambers (KMF240, Binder) for 48 h under controlled humidity and temperature conditions to promote hatching.

Hatched BSF neonates were collected and weighed on a precision scale. Neonates were reared in plastic containers and fed a nursery diet (42% chicken feed [Purina, Start & Grow] and 58% water at 35°C). Each replicate contained 50,000 neonates by weight and 1700 g of nursery diet. Replicates were placed in the incubating chambers for 96 h (3 days) under controlled humidity and temperature conditions.

After 4 days, BSF larvae were manually separated from the remaining chicken feed and combined into one sample. Then, larvae were reared in plastic containers (ULINE), each filled with 12,000 g of rearing diet maintained at 25°C–28°C. The diet consisted of a proprietary blend of cattle rumen, process water from a cattle slaughter facility, and by‐products from local grain processing facilities. 50,000 4‐day‐old larvae were placed into each container. The rearing replicates were placed into a growing room at 29°C–32°C and 65% relative humidity. On day 10, an additional 7000 g of rearing diet was added to each replicate.

On day 12, larvae were separated from the frass in each rearing replicate container using stainless steel sieves with 3‐mm mesh. If the frass was too wet for effective sieving, larvae were manually separated. To ensure the collected frass was pathogen‐free, a thermal treatment process was developed, validated through an in‐house small‐scale trial, and subsequently applied. Untreated frass was spread in sheet pans, placed on freezer racks, and transferred to smokehouses set to 93.3°C with 100% steam and no smoke. The frass was thermally treated in the smokehouse for 5 h, and then transferred to a collection bin and stored in a cooler until further use.

PL was obtained from local commercial broiler houses. The physicochemical composition of both nutrient sources (i.e., frass and PL) is shown in Table 1.

**TABLE 1:** Chemical composition of black soldier fly larvae frass and poultry litter applied in the organic soybean and switchgrass systems in Booneville, AR.

	Treatments		
Properties	Frass	PL	*p*‐value
Moisture (%)	70.5 ± 0.2a	13.4 ± 0.2b	<0.001
pH	7.60 ± 0.03b	7.77 ± 0.03a	0.014
EC (dS m^−1^)	7.73 ± 0.15b	11.94 ± 0.15a	<0.001
TC (%)	55.5 ± 0.57a	36.8 ± 0.57b	<0.001
TN (%)	3.9 ± 0.08b	4.8 ± 0.08a	0.002
TC (%)^a^	16.4 ± 0.39b	31.89 ± 0.39a	<0.001
TN (%)^a^	1.17 ± 0.07b	4.15 ± 0.07a	<0.001
N‐NO_3_^−^ (mg kg^−1^)	0.74 ± 19.7b	303.2 ± 19.7a	0.001
N‐NH_4_^+^ (mg kg^−1^)	2077 ± 116a	2054 ± 116a	0.891
P (%)	1.25 ± 0.02b	1.49 ± 0.02a	0.001
K (%)	2.26 ± 0.03b	3.29 ± 0.03a	<0.001
Ca (%)	0.13 ± 0.03b	1.88 ± 0.03a	<0.001
Mg (%)	0.48 ± 0.01b	0.67 ± 0.01a	<0.001
S (%)	1.20 ± 0.01a	1.14 ± 0.01b	0.049
Cu (mg kg^−1^)	8.3 ± 0.6b	96.5 ± 0.6a	<0.001
Fe (mg kg^−1^)	228 ± 35a	337 ± 35a	0.093
Mn (mg kg^−1^)	42.1 ± 2.7b	462.2 ± 2.7a	<0.001
Zn (mg kg^−1^)	73.7 ± 2.7b	432.3 ± 2.7a	<0.001
B (mg kg^−1^)	24.9 ± 2.1b	77.7 ± 2.1a	<0.001
Al (mg kg^−1^)	52.8 ± 15.3b	616.7 ± 15.3a	<0.001
As (mg kg^−1^)	0.53 ± 0.15a	0.87 ± 0.15a	0.184
Cd (mg kg^−1^)	0.03 ± 0.02b	0.22 ± 0.02a	0.002
Cr (mg kg^−1^)	3.83 ± 0.12b	4.48 ± 0.12a	0.020
Na (mg kg^−1^)	7617 ± 141b	9767 ± 141a	0.001
Pb (mg kg^−1^)	0.25 ± 0.03b	0.83 ± 0.03a	0.002

*Note*: Means followed by the same letter within a row do not differ (*p *> 0.05).

Abbreviations: EC, electrical conductivity; N‐NH_4_^+^, ammonium‐N; N‐NO_3_^−^, nitrate‐N; PL, poultry litter; TC, total C; TN, total N.

^a^ Fresh matter basis.

### 2.3 Site establishment and management

Four soybean rows (Natural Soybean and Grain Alliance, NSGA 52 Ci variety) were planted with a Great Plains YP‐425 planter (Great Plains Ag.) with a 75‐cm row spacing at a seeding rate of 398,132 seeds ha^−1^ in late April. Soybean plots were tilled before planting to control weeds, using a rotary tiller (Mashcio SC300). Irrigated conditions using a drip irrigation system (1.9‐cm polyethylene tubing with emitters every 30.5 cm) were deployed when <2.54 cm of rainfall per week occurred from the cotyledon emergence stage until R6 (full seed). At maturity, a 1.52‐m‐long section of each of the two middle rows was manually harvested in each plot and weighed to measure grain yield. Aboveground tissue was cut with a hedge trimmer, and grain yields were determined using an Almaco SBT thresher (Almaco). Remaining residue was weighed to determine total potential forage yields following grain harvests. Soybean samples (grain and forage) were dried in a forced‐air oven at 55°C for 48 h to determine yields on a dry matter basis.

Established switchgrass cv. Alamo plots (2.4 m × 4.6 m), planted at 7 kg pure live seed ha^−1^ with 18‐cm row spacing on April 11, 2013 were used in this study. Two harvests occurred once plots accrued adequate forage mass for harvest (July 3 and September 11, 2024). During each harvest, three 0.25 m^2^ quadrats were harvested with a residential hedge trimmer (Black and Decker) to a 15‐cm stubble height. Thereafter, forage samples were oven‐dried at 55°C for 48 h to determine yields on a dry matter basis. The remaining crop biomass was removed from each plot after each harvest.

### 2.4 Plant growth and nutritional value

Plant height was measured on 15 plants per plot concurrent at 30, 60, and 90 days after planting soybean in the row crop and pasture systems. Grains and forage samples were dried at 70°C for 48 h and ground in a Wiley mill (Thomas Scientific) to a 1‐mm mesh for further nutritional value analyses. Total C and N were determined via high‐temperature combustion (Provin, 2014) using a Vario Max CN analyzer (Elementar Americas, Inc.). Plant nutrients (i.e., Ca, Mg, P, K, and S) were measured after acid digestion using nitric acid and hydrogen peroxide, and quantified via inductively coupled argon‐plasma spectrometry on an Agilent 5110 inductively coupled optical emission spectroscopy (ICP‐OES) (Agilent Technologies; Zarcinas et al., 1987). Nutrient removal was obtained by multiplying nutrient concentration by grain or forage yields. Crude protein was calculated by multiplying percent N by 6.25. Acid‐digestible fiber (ADF), neutral‐digestible fiber (NDF), and lignin were determined with an A2000 fiber analyzer (ANKOM Technologies; Van Soest et al., 1991) and a Thermolyne muffle furnace (Thermo Fisher). Hemicellulose was calculated by subtracting NDF minus ADF. Partial factor productivity (PFP), a measure of nutrient use efficiency (NUE), was calculated for each soil amendment as grain or forage mass yields divided by the applied nutrient content (Dobermann, 2007):

PFP=Y/F,

where PFP is the partial factor productivity of applied nutrient (kg of harvested product per kg of nutrient applied), *Y* is the grain or forage mass yield in the amended plots (kg ha^−1^), and *F* is the amount of nutrient applied through each treatment (kg ha^−1^).

Leaf damage was used to assess the ability of frass to enhance plant defense and protect against insect herbivory under irrigated and non‐irrigated soybean systems. The LeafByte application (Getman‐Pickering et al., 2019), based on image processing and computational geometry, was used in the field to quantify relative leaf area reduction. Leaf damage was measured at approximately 30, 60, and 90 days after planting on three plants and 10 representative, fully expanded leaves per experimental plot. Leaves were placed on a white background for better contrast and area correction, and scanned in the LeafByte application, generating a percentage of area consumed.

### 2.5 Physicochemical characterization of amendments and soils

PL and frass samples were analyzed in triplicates. Total C and N were determined by combustion using a Vario Max CN analyzer (Elementar Americas, Inc.). Soil pH and electrical conductivity (EC) were measured on a 1:10 (fresh litter:water) sample extraction (Self‐Davis & Moore, 2000). Nitrate (N‐NO_3_^−^) and ammonium (N‐NH_4_^+^) were determined on 1:10 litter:water extraction following filtration through a 0.45‐µm filter paper (Self‐Davis & Moore, 2000) by colorimetric analysis on a Skalar San++ auto‐analyzer (Skalar, Analytical B.V.). Nitrate was analyzed by Cd‐reduction (Greenberg et al., 1992), and N‐NH_4_^+^ was analyzed by salicylate‐nitroprusside (USEPA, 1979). Total metals (Al, As, Ca, Cd, Cr, Fe, K, Mg, Mn, Na, P, Pb, S, and Zn) were determined on oven‐dried insect frass and PL samples by ICP‐OES on an Agilent 5110 ICP‐OES (Agilent Technologies), after digestion with HNO_3_ and H_2_O_2_ (Zarcinas et al., 1987).

Composite soil samples (*n* = 3) from the 0‐ to 15‐cm depth were collected from each experimental unit using a push probe (2‐cm inner diameter; AMS, Inc.) prior to the initiation of the experiment and repeated after harvest (October 2024). After air drying at 55°C for 1 week, soil samples were ground and sieved to 2 mm. Soils were analyzed for total C and N via high‐temperature combustion as described above. Soil pH and EC were measured using a 1:10 soil:water suspension (Kovar & Pierzynski, 2009). Mehlich‐3 extractable nutrients (i.e., P, K, Ca, Mg, and S) were determined using a 1:10 soil:extractant ratio (Mehlich, 1984) and analyzed using ICP‐OES 5110 (Agilent Technologies). Soil nitrate (NO_3_‐N) was extracted using a 2 M KCl solution and analyzed by Cd‐reduction (Greenberg et al., 1992), whereas KCl‐extractable ammonium (KCl‐NH_4_‐N) was analyzed by the salicylate‐nitroprusside method (USEPA, 1979), both colorimetrically on a Skalar San++ auto‐analyzer.

Soil samples for soil enzymes’ activity (β‐glucosidase [BG], acid phosphatase [AcP], and fluorescein diacetate [FDA] hydrolysis) were collected (one per plot, only for soybean) using a push probe, which was cleaned with 70% isopropyl alcohol between plots. Soil enzymes were analyzed for the CT, low, and high frass treatments. Samples were stored in a cooler and shipped the same day to the USDA‐ARS Coastal Plain Soil, Water, and Plant Conservation Research Center Soil Microbiology laboratory (Florence, SC). BG and AcP activities were determined using a colorimetric assay as described in Tabatabai (1994). *N*‐acetyl‐β‐d‐glucosaminidase (NAG) was determined using a similar method as described by Parham and Deng (2000). Overall microbial activity was measured via FDA hydrolysis as described by Prosser et al. (2011). For all assays, 1 g of air‐dried, sieved (<2 mm) soil was incubated at 37°C with their appropriate substrate. Incubation duration was 1 h for BG, AcP, and NAG and 3 h for FDA. After incubation, samples were filtered through Whatman No. 2 filter paper and absorbances measured at 405 nm (BG, AcP, and NAG) or 490 nm (FDA) on a Shimadzu UV‐1280 spectrophotometer (Shimadzu Scientific Instruments Inc.). All enzyme activities were assayed in duplicate. Auto‐hydrolysis was measured by running a CT sample alongside the duplicate soil samples, to which substrate was added post‐incubation and used to correct final enzymatic activity rates (Acosta‐Martinez et al., 2007).

### 2.6 Calculation and statistical analysis

Analysis of variance was performed to compare chemical composition of BSF larvae frass and PL, as well as to assess the effects of soil amendments and application rates on soil chemical properties, forage nutritional value, forage mass, and NUE (hypotheses 1 and 2). For switchgrass, soil amendments were considered as a fixed effect, harvest as repeated measures, and replicates as random. As for soybean, soil and plant health response variables were analyzed in a completely randomized block design, considering irrigation (irrigated and non‐irrigated) as the whole plot, amendments as the split‐plot, and replicates as random.

For plant heights and leaf damage measured at 30, 60, and 90 days after treatment application, soil amendments and irrigation were considered the fixed effect, measurement dates as repeated measures, and replicates as random. Mean separation was performed using Fisher's least significant difference and Type I error rate of 5%. Statistical analyses were performed using the SAS (SAS 9.4) software (SAS Institute, 2016).

## 3 RESULTS AND DISCUSSION

### 3.1 Poultry litter versus frass chemical composition

PL had higher pH, EC, total N, and nearly 400 times greater NO_3_^−^‐N than frass (*p* < 0.05; Table 1; dry matter basis). Ammonium levels were similar between frass and PL (∼2000 mg kg^−1^; *p* > 0.05). PL also had greater P, K, Ca, and Mg levels than frass, much higher micronutrient (Cu, Fe, Mn, Zn, and B) concentrations, as well as heavy metals (seven times more Cd, 17% more Cr, and three times more Pb) and other potentially toxic elements (12 times more Al and 28% greater Na; *p* < 0.05). In turn, frass had higher moisture (71%, compared to 13% for PL), 51% higher total C (TC) (dry matter basis), and 5% higher S (*p* < 0.05). Despite the lower nutrient concentrations relative to PL, frass N, P, and K concentrations (4%, 1.3%, and 2.3% in a dry matter basis, respectively) were similar to other BSF larvae frass reported in the literature (Beesigamukama et al., 2022; Lopes et al., 2022; Song et al., 2021) and illustrated frass’ usefulness as an organic fertilizer source. Differences in chemical composition and the increased moisture content in frass reflect the BSF larvae rearing diet (e.g., grain processing by‐products and industrial wastewater), as well as amendments and growth‐promoting supplements in the poultry feed (Amorim et al., 2024).

### 3.2 Soil nutrients and microbial activity as affected by organic fertilizers

Irrigated soybean systems had generally greater soil fertility than non‐irrigated; yet, soil chemical properties were mostly unaffected by soil amendments (Table S1). High frass increased (*p* < 0.05) soil pH (7.08) compared to LF and PL (6.45–6.65), not differing from the CT in irrigated systems. Additionally, LF and HF increased (*p *< 0.05) soil NO_3_^−^‐N levels in non‐irrigated systems (13.6 and 15.1 mg kg^−1^, respectively) relative to the CT (6.4 mg kg^−1^), not differing from PL (10.9 mg kg^−1^). As for irrigated soybean systems, soil NO_3_^−^‐N in LF and HF were similar to the CT, decreasing 37% under PL (8.5 mg kg^−1^; Table S1). These increased NO_3_^−^‐N levels under frass treatments were unexpected, considering the 400 times greater NO_3_^−^‐N in raw PL (Table 1). Nevertheless, PL mostly consists of organic‐N (95% of total N), and 50%–60% of organic‐N becomes available through mineralization in the first year (Ashworth, Chastain, et al., 2020). In the current study, it is possible that nitrification from frass occurred faster than PL, which may explain the higher soil NO_3_^−^‐N levels, particularly under non‐irrigated systems. Watson et al. (2021) reported increased soil NO_3_^−^‐N from frass in short‐term incubation studies (25–92 mg kg^−1^), particularly at greater application rates and without nitrification inhibitors. Yet, the levels reported in the current study can be considered low to ideal for agricultural soils (13–17 mg kg^−1^; Schmitt & Randall, 1994) and do not pose risk to environmental quality. Additional work is needed to evaluate the organic–inorganic transformation of frass to better understand nutrient dynamics in the production environment.

For switchgrass, the application of LF and PL increased total soil N relative to the CT (Table S2; *p* < 0.05), with HF showing an intermediate value. Soil pH, EC, soil organic C (SOC) and all other nutrients were unaffected by soil amendment type or rate. Soil NO_3_^−^‐N was not detected in switchgrass soils receiving either frass or PL (data not shown). This general lack of effects from soil amendments can be attributed to the short duration of the experiment (i.e., 5 months from treatment application to harvest/soil sampling). After 2 years of application of yellow mealworm (*Tenebrio molitor* L.) larvae frass (3% N, 2% P, and 2% K), Ashworth et al. (2025) observed substantial soil fertility improvements in bermudagrass [*Cynodon dactylon* (L.) Pers.] forage systems. These authors reported twofold and threefold increases in SOC and N stocks from frass compared to ammonium nitrate, and 50 kg ha^−1^ increase in plant‐available P and K compared to losses of 25–50 kg ha^−1^ in plots receiving PL. Here, increases in soil N from LF were noted relative to the CT; however, more consistent improvements in soil organic matter and nutrient availability in row crop and forage systems may be observed after additional years of evaluation. As such, for the 2024 growing season, we partially reject our first hypothesis of enhanced soil fertility from frass.

The BG enzyme and FDA activity were unaffected by soil amendments (Table 2; *p* > 0.05) or irrigation (data not shown), demonstrating frass application does not disrupt overall microbial activity in soils compared to the unamended systems. However, NAG, used as a measure of chitinase activity, decreased in LF compared to the CT (Table 2; *p* < 0.05). The same trend was noted for AcP, indicating that LF reduced chitinase and AcP activities in soil. However, HF had intermediate values of NAG and AcP and did not differ from the CT. These findings contrast with the initial hypothesis that BSF larvae frass would result in enhanced nutrient cycling and microbial activity in soils (Esteves et al., 2022; Nurfikari et al., 2024). Specifically, we expected chitin in BSF larvae frass would enhance NAG activity compared to the CT, but changes in soil pH and NO_3_^−^‐N levels (Table S1) may have affected enzyme activity. This is supported by Li et al. (2019) who demonstrated a positive relationship between NAG activity and increasing soil N. The transitory nature of bacterial chitinolytic activity may have also played a confounding role in the expected versus observed results. Studies by Hui et al. (2020) and Jacquiod et al. (2013) both demonstrated a peak in chitinase activity within 10 days of chitin amendment, with chitinase activity dropping precipitously over the following 10–30 days. These findings indicate that further study into temporal trends following frass application on microbial structure and function, particularly as it pertains to chitin availability, is warranted.

**TABLE 2:** Soil enzymes’ activity (0–15 cm) as affected by soil amendments (LF, low frass rate; HF, high frass rate) in an organic soybean cropping system in Booneville, AR, during the 2024 growing season, averaged by irrigation (non‐irrigated and irrigated).

	Soil enzymes			
Treatment	BG (µmol pNP g^−1^ soil h^−1^)	NAG (µmol pNP g^−1^ soil h^−1^)	AcP (µmol pNP g^−1^ soil h^−1^)	FDA (µg fluorescein g^−1^ soil h^−1^)
Control	77.4 ± 6.7a	40.4 ± 3.4a	293.0 ± 19.6a	255.0 ± 27.3a
LF	63.8 ± 6.7a	29.4 ± 3.4b	227.1 ± 19.6b	256.2 ± 27.3a
HF	65.1 ± 6.7a	35.0 ± 3.4ab	263.5 ± 19.6ab	261.5 ± 27.3a
*p*‐value	0.114	0.009	0.003	0.622

*Note*: Means followed by the same letter within a column do not differ (*p *> 0.05).

Abbreviations: AcP, acid phosphatase; BG, β‐Glucosidase; FDA, fluorescein diacetate; NAG, *N*‐acetyl‐β‐d‐glucosaminidase; pNP, para‐nitrophenol.

### 3.3 Plant growth and enhanced pest resistance from frass

PL applications increased soybean heights by 5%–6% (86 ± 3 cm) relative to the CT (82 ± 3 cm) and frass treatments (81–82 ± 3 cm) when averaged across irrigation and days after treatment application (*p* = 0.01; data not shown). Specifically, in non‐irrigated systems, PL‐amended soybean was 5% and 8% taller than LF and HF‐amended plots, respectively, not differing from the CT (*p *< 0.05). In irrigated systems, LF increased soybean heights by 6% compared to the CT, being similar to PL and HF (61 and 58 cm, respectively). As for switchgrass, mean height was unaffected by soil amendments (*p* < 0.05; data not shown), varying only based on measurement dates (50, 115, and 84 cm at 30, 60, and 90 days after treatment application, respectively; *p* < 0.001).

**FIGURE 1:**
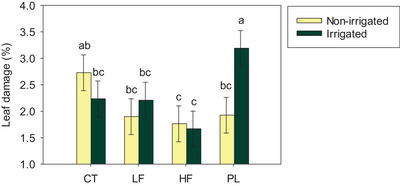
Interactive effects of irrigation (non‐irrigated and irrigated) and soil amendments (CT, control; LF, low frass rate; HF, high frass rate; PL, poultry litter) on leaf damage in an organic soybean cropping system in Booneville, AR, during the 2024 growing season. Means ± standard errors followed by the same letter do not differ (*p* > 0.05).

Leaf damage was only visible in the soybean production system, and therefore it was not evaluated in the switchgrass system. In non‐irrigated soybean, HF reduced mean leaf damage by pest insects (averaged across measurement dates) by 35% compared to the CT (Figure 1; *p* < 0.05), not differing from LF and PL. As for irrigated soybean, PL had the highest leaf damage, while LF and HF had 31% and 48% lower damage than PL, respectively, thus leading us to accept our hypothesis of enhanced plant resistance from frass. Since changes in overall soil microbial activity from frass were not observed, possible causes of reduced leaf damage in HF–soybean plots include enhanced tolerance to herbivory triggered by chitin (Poveda, 2021), attraction of more natural enemies, or changes in soil microbiome (Barragán‐Fonseca et al., 2022; Kisaakye et al., 2024). However, these mechanisms were not assessed in the present study, and thus future research should elucidate how frass from various insect species affects complex soil–microbe–plant interactions. Our findings illustrate, for the first time under field conditions, frass improvements in plant resistance to herbivory and showcase frass potential as a natural pesticide for non‐irrigated organic soybean systems.

### 3.4 Crop yields, quality, and nutrient use efficiency in organic soybean and switchgrass

**FIGURE 2:**
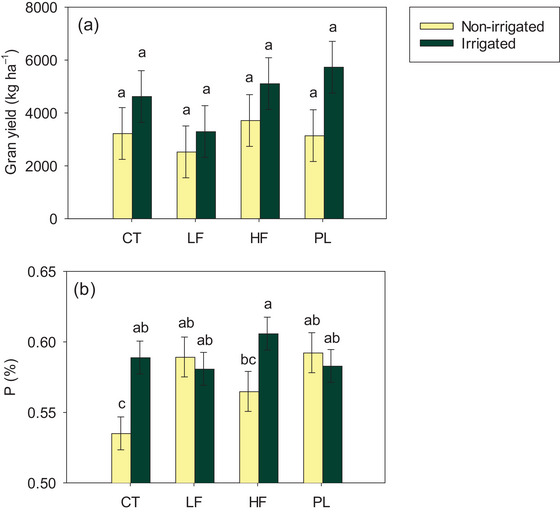
Interactive effects of irrigation (non‐irrigated and irrigated) and soil amendments (CT, control; LF, low frass rate; HF, high frass rate; PL, poultry litter) on soybean grain yield (a) and P concentration (b) in an organic soybean cropping system in Booneville, AR, during the 2024 growing season. Means ± standard errors followed by the same letter within a panel do not differ (*p* > 0.05).

Irrigated soybean had 49% greater grain yields, 59% greater K removal, and higher P concentration in grains than non‐irrigated systems, which in turn had higher total C and N concentrations than irrigated soybean (*p *< 0.05; Table S3). Most macro‐ (Table S3) and micronutrients (data not shown) concentrations were unaffected by soil amendments or the interactive effects between irrigation × amendment. Nevertheless, a trend of increased grain yields in non‐irrigated‐HF (3717 kg ha^−1^) soybean systems relative to the CT was observed (Figure 2a; CT yield: 3226 kg ha^−1^), suggesting a positive effect from frass under non‐irrigated organic soybean systems. In turn, PL in irrigated systems seemed to increase grain yields (5732 kg ha^−1^) compared to LF (3299 kg ha^−1^) and CT (4624 kg ha^−1^). Grain P concentration in HF‐irrigated plots (6%) was 7% higher than non‐irrigated (5.6%), and 13% greater than the non‐irrigated CT (5.4%; Figure 2b; *p* < 0.05), demonstrating nutrient dynamics differ under irrigated and dryland conditions for these two manure sources.

**FIGURE 3:**
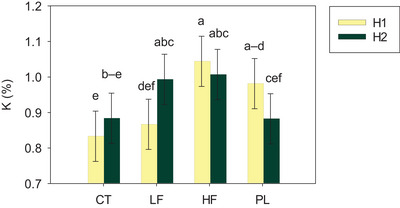
Interactive effects of harvest (H1, harvest 1; H2, harvest 2) and soil amendments (LF, low frass rate; HF, high frass rate; PL, poultry litter) on forage K concentration (b) in an organic pasture system in Booneville, AR, during the 2024 growing season. Means ± standard errors followed by the same letter do not differ (*p* > 0.05).

Switchgrass biomass and nutritional value were mostly unaffected by soil amendments, varying with harvest (Table S4). Yet, the mean biomass values of LF‐ (9.5 Mg ha^−1^) and HF‐amended plots (8.6 Mg ha^−1^; Table S4) were similar or higher than those receiving >168 kg N ha^−1^ as ammonium nitrate in previous work (Ashworth, Moore, et al., 2020), suggesting that frass can potentially replace standard synthetic fertilizers with no yield losses. In the current study, cumulative forage yields (harvest 1 + harvest 2) ranged between 17.2 Mg ha^−1^ for HF and 18.9 Mg ha^−1^ for LF (Table 3), but did not differ from the unamended CT (17.3 Mg ha^−1^; *p *= 0.8483). Mean forage mass was 52% greater in the first harvest, with greater TC and 50% higher K removal (Table S4). Forage K concentrations were affected by harvest × soil amendments (Figure 3; *p* < 0.05). Specifically, HF increased K concentration in switchgrass biomass by 25% relative to CT, and by 20% compared to LF in the first harvest, not differing from PL. In the second harvest, K concentrations in LF‐amended switchgrass increased by 14%, being similar to K concentrations in HF‐ and PL‐amended plots. These findings aligned with previous observations of increased K uptake in frass‐amended bermudagrass (Ashworth et al., 2025) and showcase frass as an excellent K source for pasture systems, which is particularly relevant for drought and stress tolerance, carbohydrate storage, and greater winter survival and recovery (Marschner, 2012; D. L. Robinson, 1985). Despite the enhanced P concentration in soybean and K concentration in switchgrass relative to the CT, we reject our hypothesis of enhanced nutritional value from frass compared to PL.

**TABLE 3:** Nutrient use efficiency as affected by soil amendments (LF, low frass rate; HF, high frass rate; PL, poultry litter) in organic soybean and pasture systems in Booneville, AR, during the 2024 growing season.

			Nutrient use efficiency					
Treatment	Rate (Mg ha^−1^)	Yield (kg ha^−1^)	N applied (kg ha^−1^)	N PFP (kg kg^−1^)	P applied (kg ha^−1^)	P PFP (kg kg^−1^)	K applied (kg ha^−1^)	K PFP (kg kg^−1^)
			**Soybean**					
LF	5.6	2915	66	44 ± 5a	21	141 ± 16a	37	77 ± 9a
HF	11.2	4414	131	34 ± 5a	41	107 ± 16a	75	59 ± 9a
PL	5.6	4438	232	19 ± 5b	72	61 ± 16b	160	28 ± 9b
*p*‐value				0.002		0.004		0.002
			**Pasture**					
LF	5.6	18,894	66	159 ± 10a	21	915 ± 49a	37	276 ± 17a
HF	11.2	17,167	131	78 ± 10b	41	416 ± 49b	75	135 ± 17b
PL	5.6	17,621	232	48 ± 10b	72	245 ± 49c	160	106 ± 17b
*p*‐value				0.002		0.002		0.001

*Note*: Means followed by the same letter within a column do not differ (*p *> 0.05).

Abbreviation: PFP, partial factor productivity.

Understanding NUE of novel organic fertilizer sources is key to promoting their effective utilization, enhanced productivity, and environmental quality. Low frass had the highest partial factor productivity (PFP) in both soybean and switchgrass systems (Table 3; *p *< 0.05). In soybean, LF had two times greater N and P PFP and three times greater K PFP than PL as a result of similar grain yields using 72% less N, 71% less P, and 77% less K than that applied via PL (Table 2). NUE from HF was similar to LF in the soybean systems, owing to numerically higher yields (Figure 2a) and a two times greater application rate than LF. As for switchgrass, LF had three, four, and three times higher N, P, and K PFP than PL, respectively, with HF showing intermediate efficiency between the two sources (Table 3; *p* < 0.05). Together, these findings showcased the potential of low frass rates for organic production systems, leading to grain and forage yields comparable to PL, but using much lower nutrient inputs.

**FIGURE 4:**
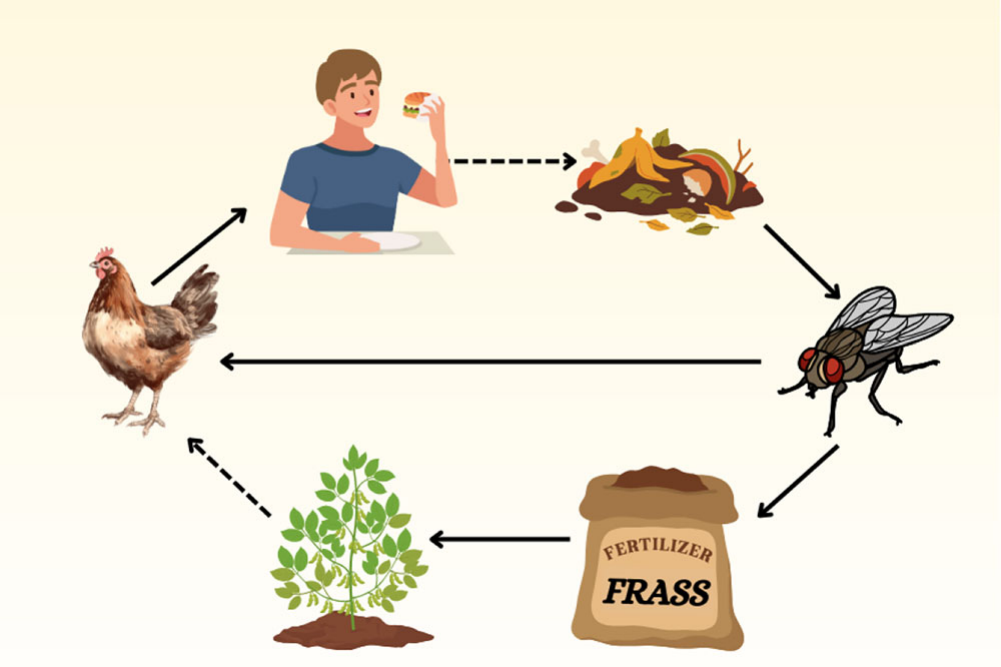
Insect frass use supports a circular economy by allowing for waste upcycling as insect feed, reduced landfill waste accumulation and dependence on annual grains (denoted by dashed arrows), and enhanced crop growth and nutritional value, potentially displacing synthetic fertilizers and reducing the environmental footprint of row crop agriculture.

This field study comparing BSF larvae frass and PL showcased the potential of frass to advance nutrient recovery by rearing insects on agricultural waste and producing a value‐added nutrient source and a plant growth promoter (Figure 4) for major agricultural crops and forage‐biomass species. While changes in soil nutrients and microbial activity were minimal, likely as a result of the short evaluation period, improvements in grain and forage nutritional value and yields from frass were similar to PL, a standard organic fertilizer for regional row crops and pastures. As BSF larvae frass was generally less nutrient‐enriched than PL, application of fresh mass‐based rates for frass treatments resulted in lower nutrient inputs; however, similar grain and forage yields evidenced enhanced NUE from LF. Lower leaf damage by pest insects was unraveled as another beneficial trait of frass, although the mechanisms of this greater plant resistance were not elucidated in the current study. Therefore, further research is needed to support frass use as pesticide/biostimulator, allowing for proper product certification and additional revenue for insect producers. In summary, our findings demonstrated frass as a multifunctional soil amendment, which can improve whole system's sustainability and foster a circular economy by (1) recycling agricultural, industry, and household waste as insect feed and reducing waste accumulation in landfills, (2) allowing for a sustainable protein source for the poultry sector, potentially supporting the partial displacement of annual grains in animal diets, and (3) providing a nutrient source for various cropping systems, thus halting reliance on synthetic fertilizers and reducing pesticide use for farmers. Farm products will feed animals or humans, who in turn will keep generating waste to be used as insect feed (Figure 4).

## 4 CONCLUSIONS

Insect manure or “frass” is a promising amendment for organic and conventional systems, with 12 times greater concentrations of heavy metals and potentially toxic elements than PL. For the first time, insect manure “frass” was showcased as a multifunctional amendment to enhance NUE and pest resistance at the field scale. High BSF larvae frass (11.2 Mg ha^−1^) reduced soybean leaf damage by 48% and 35% relative to PL‐irrigated and the non‐irrigated CT treatments, and increased P and K concentrations in soybean grains and switchgrass biomass, respectively. Low frass (5.6 Mg ha^−1^) had two times greater N and P use efficiency and three times greater K use efficiency than PL in soybean, and three to four times higher N, P, and K use efficiency than PL in switchgrass. In the next phase of our research, we will examine nutrient release from various frass sources, as well as changes in soil microbial diversity, and how this impacts biomass and crop yields. Here, we demonstrated frass as a powerful organic fertilizer that improves plant nutrition and helps protect crops, while closing nutrient loops and creating value‐added products for the fast‐growing insect farming industry.

## AUTHOR CONTRIBUTIONS

**Helen C. S. Amorim**: Data curation; formal analysis; software; writing—original draft; writing—review and editing. **Amanda J. Ashworth**: Conceptualization; investigation; methodology; resources; writing—original draft; writing—review and editing. **Thomas F. Ducey**: Investigation; methodology; writing—original draft; writing—review and editing. **Valerie B. Brewer‐Gunsaulis**: Methodology; resources; supervision; writing—review and editing. **Gerson L. Drescher**: Supervision; validation; writing—original draft; writing—review and editing. **Phillip R. Owens**; Investigation; resources; supervision; validation; writing—review and editing. **Alana H. Patterson**: Methodology; resources; writing—review and editing. **Giovanna De Blasis**: Methodology; resources; writing—review and editing. **Iris van Straaten**: Methodology; resources; writing—review and editing.

## CONFLICT OF INTEREST STATEMENT

The authors declare no conflicts of interest.

## Supporting information



The Supplemental Material file contains 4 tables, including information on soil chemical properties (0‐15 cm) and grain/biomass yields and nutrient concentration as affected by irrigation and soil amendments (LF, low frass rate; HF, high frass rate; PL, poultry litter) in organic soybean and switchgrass systems in Booneville, AR, during the 2024 growing season.
