# Supplementary material for: Recycling waste via insect agriculture: Frass impacts on soil and plant health
Source: J Environ Qual. 2025 Sep 25;54(6):1457–69. doi: 10.1002/jeq2.70089 (PMC12593254; doi:10.1002/jeq2.70089)
Supplement: Supplementary file 1 — The Supplemental Material file contains 4 tables, including information on soil chemical properties (0‐15 cm) and grain/biomass yields and nutrient concentration as affected by irrigation and soil amendments (LF, low frass rate; HF, high frass rate; PL, poultry litter) in organic soybean and switchgrass systems in Booneville, AR, during the 2024 growing season. [file JEQ2-54-1457-s001.docx]

**Supplemental Table 1.** Soil properties (0-15 cm) as affected by irrigation and soil amendments (LF, low frass rate; HF, high frass rate; PL, poultry litter) in an organic soybean cropping system in Booneville, AR, during the 2024 growing season.

| **Irrigation** | **Treatment**† | **Soil Properties**‡ | | | | | | | | | | |
| --- | --- | --- | --- | --- | --- | --- | --- | --- | --- | --- | --- | --- |
|  |  | **SOC** | **N** | **N-NH_4_^+^** | **N-NO_3_^-^** | **pH** | **EC** | **Ca** | **K** | **Mg** | **P** | **S** |
|  |  | % | | mg kg^-1^ | |  |  | mg kg^-1^ | | | | |
| Non-irrigated | Control | 1.31 ± 0.17 a⸹ | 0.15 ± 0.01 a | 9.59 ± 1.07 a | 6.38 ± 3.18 c | 6.61 ± 0.09 b | 62.5 ± 9.1 a | 411 ± 41 a | 12.9 ± 4.4 a | 40.8 ± 6.9 a | 9.85 ± 1.9 a | 15.4 ± 5.3 a |
|  | LF | 1.49 ± 0.17 a | 0.16 ± 0.01 a | 10.01 ± 1.07 a | 13.57 ± 3.18 a | 6.47 ± 0.09 b | 68.2 ± 9.1 a | 390 ± 41 a | 19.5 ± 4.4 a | 38.1 ± 6.9 a | 5.92 ± 1.9 a | 6.9 ± 5.3 a |
|  | HF | 1.45 ± 0.17 a | 0.16 ± 0.01 a | 10.03 ± 1.07 a | 15.12 ± 3.18 a | 6.45 ± 0.09 b | 59.9 ± 9.1 a | 383 ± 41 a | 12.0 ± 4.4 a | 35.9 ± 6.9 a | 6.40 ± 1.9 a | 7.5 ± 5.3 a |
|  | PL | 1.63 ± 0.17 a | 0.16 ± 0.02 a | 11.70 ± 1.07 a | 10.95 ± 3.53 abc | 6.46 ± 0.09 b | 71.2 ± 9.1 a | 419 ± 41 a | 18.9 ± 4.4 a | 39.7 ± 6.9 a | 7.95 ± 1.9 a | 8.1 ± 5.3 a |
| Irrigated | Control | 1.59 ± 0.17 a | 0.16 ± 0.01 a | 9.82 ± 1.31 a | 13.40 ± 3.18 a | 6.98 ± 0.09 a | 81.2 ± 9.1 a | 606 ± 41 a | 27.2 ± 4.4 a | 54.2 ± 6.9 a | 23.76 ± 2.3 a | 37.2 ± 5.3 a |
|  | LF | 1.52 ± 0.17 a | 0.16 ± 0.01 a | 9.81 ± 1.31 a | 16.70 ± 3.18 a | 6.49 ± 0.11 b | 75.9 ± 9.1 a | 572 ± 41 a | 21.2 ± 4.4 a | 65.6 ± 6.9 a | 16.44 ± 2.3 a | 40.3 ± 5.3 a |
|  | HF | 1.44 ± 0.17 a | 0.16 ± 0.01 a | 11.67 ± 1.31 a | 12.04 ± 3.18 ab | 7.08 ± 0.09 a | 90.3 ± 9.1 a | 615 ± 41 a | 21.6 ± 4.4 a | 57.9 ± 6.9 a | 18.67 ± 2.1 a | 36.2 ± 5.3 a |
|  | PL | 1.59 ± 0.17 a | 0.16 ± 0.01 a | 12.45 ± 1.31 a | 8.50 ± 3.53 bc | 6.65 ± 0.09 b | 89.9 ± 9.1 a | 557 ± 41 a | 28.1 ± 4.4 a | 60.7 ± 6.9 a | 18.25 ± 2.1 a | 42.4 ± 5.3 a |
| *p-value* |  | *0.69* | *0.90* | *0.81* | *0.04* | *0.02* | *0.39* | *0.48* | *0.16* | *0.78* | *0.64* | *0.12* |
| †LF, low frass rate; HF, high frass rate; PL, poultry litter;  ‡SOC, soil organic C; N-NH_4_^+^, ammonium-N; N-NO_3_^-^, nitrate-N; EC, electrical conductivity;  ⸹ Means followed by the same letter do not differ (*p* > 0.05). | | | | | | | | | | | | |

**Supplemental Table 2.** Soil properties (0-15 cm) as affected by soil amendments (LF, low frass rate; HF, high frass rate; PL, poultry litter) in an organic switchgrass system in Booneville, AR, during the 2024 growing season.

| **Treatment**† | **Soil Properties**‡ | | | | | | | | | |
| --- | --- | --- | --- | --- | --- | --- | --- | --- | --- | --- |
|  | **SOC** | **N** | **N-NH_4_^+^** | **pH** | **EC** | **Ca** | **K** | **Mg** | **P** | **S** |
| Control | 1.64 ± 0.08 a⸹ | 0.15 ± 0.01 b | 11.89 ± 1.04 a | 6.17 ± 0.06 a | 35.5 ± 2.6 a | 562 ± 38 a | 24.5 ± 1.9 a | 27.0 ± 2.3 a | 12.2 ± 2.2 a | 25.5 ± 1.9 a |
| LF | 1.88 ± 0.08 a | 0.17 ± 0.01 a | 13.27 ± 0.85 a | 6.15 ± 0.06 a | 36.3 ± 2.6 a | 592 ± 38 a | 28.1 ± 1.9 a | 29.8 ± 2.3 a | 17.2 ± 2.2 a | 25.2 ± 1.9 a |
| HF | 1.83 ± 0.08 a | 0.16 ± 0.01 ab | 12.36 ± 0.85 a | 6.09 ± 0.06 a | 38.6 ± 2.6 a | 581 ± 38 a | 28.5 ± 2.2 a | 28.3 ± 2.3 a | 14.6 ± 2.2 a | 27.6 ± 1.9 a |
| PL | 1.79 ± 0.08 a | 0.17 ± 0.01 a | 14.59 ± 0.85 a | 6.15 ± 0.06 a | 35.8 ± 2.6 a | 623 ± 38 a | 26.5 ± 2.2 a | 31.3 ± 2.8 a | 15.0 ± 2.7 a | 24.5 ± 2.4 a |
| *p-value* | *0.26* | *0.03* | *0.27* | *0.64* | *0.81* | *0.73* | *0.23* | *0.60* | *0.51* | *0.76* |
| †LF, low frass rate; HF, high frass rate; PL, poultry litter;  ‡SOC, soil organic C; N-NH_4_^+^, ammonium-N; N-NO_3_^-^, nitrate-N; EC, electrical conductivity;  ⸹ Means followed by the same letter do not differ (*p* > 0.05). | | | | | | | | | | |

**Supplemental Table 3.** Soybean grain yield and chemical composition as affected by irrigation and soil amendments (LF, low frass rate; HF, high frass rate; PL, poultry litter) in an organic soybean cropping system in Booneville, AR, during the 2024 growing season.

| **Main Effect†** | **Yield** | **Chemical Composition‡** | | | | | | | | | |
| --- | --- | --- | --- | --- | --- | --- | --- | --- | --- | --- | --- |
|  |  | **TC** | **TN** | **N removal** | **K** | **K removal** | **P** | **P removal** | **Ca** | **Mg** | **S** |
|  | kg ha^-1^ | % | | kg N ha^-1^ | % | kg K ha^-1^ | % | kg P ha^-1^ | % | | |
| Non-irrigated | 3,154 ± 489 b⸹ | 52.1 ± 0.12 a | 7.3 ± 0.09 a | 230 ± 35 a | 2.0 ± 0.08 a | 63 ± 10 b | 0.57 ± 0.01 b | 18 ± 4 a | 0.32 ± 0.01 a | 0.25 ± 0.01 a | 0.35 ± 0.01 a |
| Irrigated | 4,692 ± 489 a | 50.5 ± 0.12 b | 6.8 ± 0.09 b | 320 ± 35 a | 2.1 ± 0.08 a | 100 ± 10 a | 0.59 ± 0.01 a | 28 ± 3 a | 0.33 ± 0.01 a | 0.25 ± 0.01 a | 0.34 ± 0.01 a |
| *p-value* | *0.041* | *0.001* | *0.021* | *0.105* | *0.394* | *0.027* | *0.036* | *0.093* | *0.153* | *0.502* | *0.4477* |
| Control | 3,925 ± 692 a | 51.3 ± 0.16 a | 7.1 ± 0.08 a | 277 ± 49 | 2.2 ± 0.08 a | 84 ± 14 a | 0.56 ± 0.01 a | 22 ± 4 a | 0.33 ± 0.01 a | 0.26 ± 0.01 a | 0.35 ± 0.01 a |
| LF | 2,914 ± 692 a | 51.3 ± 0.16 a | 7.1 ± 0.08 a | 207 ± 49 | 2.1 ± 0.08 a | 59 ± 14 a | 0.59 ± 0.01 a | 18 ± 4 a | 0.31 ± 0.01 a | 0.24 ± 0.01 b | 0.34 ± 0.01 a |
| HF | 4,414 ± 692 a | 51.3 ± 0.16 a | 6.9 ± 0.08 a | 308 ± 49 | 2.0 ± 0.08 a | 90 ± 14 a | 0.59 ± 0.01 a | 28 ± 4 a | 0.32 ± 0.01 a | 0.25 ± 0.01 ab | 0.34 ± 0.01 a |
| PL | 4,438 ± 692 a | 51.4 ± 0.16 a | 6.9 ± 0.08 a | 308 ± 49 | 2.1 ± 0.08 a | 92 ± 14 a | 0.59 ± 0.01 a | 25 ± 4 a | 0.33 ± 0.01 a | 0.24 ± 0.01 b | 0.34 ± 0.01 a |
| *p-value* | *0.324* | *0.956* | *0.364* | *0.374* | *0.5095* | *0.316* | *0.099* | *0.413* | *0.779* | *0.022* | *0.5986* |
| † LF, low frass rate; HF, high frass rate; PL, poultry litter;  ‡ TC, total C; TN, total N;  ⸹ Means followed by the same letter within a column do not differ (*p* > 0.05). | | | | | | | | | | | |

**Supplemental Table 4.** Forage yield and chemical composition as affected by harvest (averaged by soil amendments) and soil amendments across harvests (LF, low frass rate; HF, high frass rate; PL, poultry litter) in an organic switchgrass system in Booneville, AR, during the 2024 growing season.

| **Main Effect†** | **Yield** | **Chemical Composition‡** | | | | | | | | | |
| --- | --- | --- | --- | --- | --- | --- | --- | --- | --- | --- | --- |
|  |  | **TC** | **CP** | **N removal** | **K** | **K removal** | **P** | **P removal** | **Ca** | **Mg** | **S** |
|  | kg ha^-1^ | % | | kg N ha^-1^ | % | kg K ha^-1^ | % | kg P ha^-1^ | % | | |
| 1^st^ Harvest | 10,694 ± 693 a | 43.3 ± 0.15 a | 3.3 ± 0.10 b | 57 ± 4 a | 0.93 ± 0.06 a | 99 ± 6 a | 0.15 ± 0.01 a | 16 ± 1 a | 0.21 ± 0.02 a | 0.13 ± 0.01 b | 0.09 ± 0.01 b |
| 2^nd^ Harvest | 7,040 ± 693 b | 41.8 ± 0.15 b | 4.9 ± 0.10 a | 55 ± 4 a | 0.94 ± 0.06 a | 66 ± 6 b | 0.17 ± 0.01 a | 12 ± 1 b | 0.18 ± 0.02 a | 0.15 ± 0.01 a | 0.12 ± 0.01 a |
| *p-value* | *0.004* | *< 0.001* | *< 0.001* | *0.756* | *0.610* | *0.003* | *0.182* | *0.042* | *0.071* | *0.001* | *< 0.001* |
| Control | 8,628 ± 853 a | 42.5 ± 0.18 a | 4.0 ± 0.13 a | 51 ± 5 a | 0.86 ± 0.07 a | 72 ± 8 a | 0.13 ± 0.04 a | 10 ± 2 a | 0.18 ± 0.02 a | 0.14 ± 0.01 a | 0.11 ± 0.01 a |
| LF | 9,447 ± 853 a | 42.6 ± 0.18 a | 4.1 ± 0.13 a | 60 ± 5 a | 0.93 ± 0.07 a | 86 ± 8 a | 0.17 ± 0.04 a | 16 ± 2 a | 0.16 ± 0.02 a | 0.15 ± 0.01 a | 0.11 ± 0.01 a |
| HF | 8,583 ± 853 a | 42.3 ± 0.18 a | 4.3 ± 0.13 a | 57 ± 5 a | 1.03 ± 0.07 a | 88 ± 8 a | 0.18 ± 0.04 a | 15 ± 2 a | 0.20 ± 0.02 a | 0.14 ± 0.01 a | 0.11 ± 0.01 a |
| PL | 8,811 ± 853 a | 42.7 ± 0.18 a | 4.2 ± 0.13 a | 57 ± 5 a | 0.93 ± 0.07 a | 84 ± 8 a | 0.17 ± 0.04 a | 15 ± 2 a | 0.22 ± 0.02 a | 0.15 ± 0.01 a | 0.11 ± 0.01 a |
| *p-value* | *0.853* | *0.329* | *0.641* | *0.647* | *0.095* | *0.540* | *0.263* | *0.624* | *0.500* | *0.352* | *0.496* |
| † LF, low frass rate; HF, high frass rate; PL, poultry litter;  ‡ TC, total C; CP, crude protein;  ⸹ Means followed by the same letter within a column do not differ (*p* > 0.05). | | | | | | | | | | | |
